# Implementation of Traffic Light System on Food Sold at Brockfield House Medium Secure Unit to Help Improve Healthy Food Options

**DOI:** 10.1192/bjo.2022.163

**Published:** 2022-06-20

**Authors:** Ngozi Agunwamba, David Ho, Raman Deo, Maniya Duffy, Stavroula Madenlidou, Fintan Larkin

**Affiliations:** 1 Essex Partnership University NHS Foundation Trust, Wickford Essex, United Kingdom; 2 West London Mental Health NHS Trust, London, United Kingdom

## Abstract

**Aims:**

Public Health England published a report in 2017 on Obesity in Secure Mental Health units. A key finding of the review was that not only is obesity and overweight more prevalent in the population detained within mental health secure units (with rates of up to 80% reported) than in the general population (around 60%), patients appear to be more at risk of weight gain when detained. The report found evidence that there is a high risk of weight gain following admission, stemming from the combined effects of incarceration, ease of access to high calorific food, and the potential lack of access to recommended levels of physical activity. This project aims to; 1. Implement a traffic light system on food and confectionaries sold at the shop at a Medium secure hospital. 2. Provide healthier food options at the shop by using the traffic light system as a visual aid 3. To achieve weight reduction and promote healthy lifestyle choices in patients admitted to our medium secure Forensic unit.

**Methods:**

1. Buying a new till system which is able to quantify what type of food is sold.
2. Labelling food sold using a traffic light system.
3. Calculate types of food sold following a three-month period after implementation.

**Results:**

1. Traffic light system provides a visual aid to patients in choosing healthier food.
2. Patients in our medium secure unit achieve a reduction in their weight.
3. Traffic light system can be replicated/ adopted by other secure hospitals.

**Conclusion:**

The purpose of this research is to implement a traffic light system on food sold at a shop in our medium secure unit. It is hoped that by providing visual aids, patients have a means of easily identifying healthier food options. Choosing healthier food we hope will consequently result in weight reduction and overall improved lifestyle choices.

